# Microstructural segmentation using a union of attention guided U-Net models with different color transformed images

**DOI:** 10.1038/s41598-023-32318-9

**Published:** 2023-04-07

**Authors:** Momojit Biswas, Rishav Pramanik, Shibaprasad Sen, Aleksandr Sinitca, Dmitry Kaplun, Ram Sarkar

**Affiliations:** 1 Department of Metallurgical and Material Engineering, Jadavpur University, Kolkata, West Bengal 700032 India; 2 Department of Computer Science and Engineering, Jadavpur University, Kolkata, West Bengal 700032 India; 3 Department of Computer Science and Engineering (AI-ML), Techno Main Salt Lake, Kolkata, West Bengal 700091 India; 4 Research Centre for Digital Telecommunication Technologies, Saint Petersburg Electrotechnical University “LETI”, Saint Petersburg, Russian Federation 197022; 5 Department of Automation and Control Processes, Saint Petersburg Electrotechnical University “LETI”, Saint Petersburg, Russian Federation 197022

**Keywords:** Structural materials, Computational methods

## Abstract

Metallographic images or often called the microstructures contain important information about metals, such as strength, toughness, ductility, corrosion resistance, which are used to choose the proper materials for various engineering applications. Thus by understanding the microstructures, one can determine the behaviour of a component made of a particular metal, and can predict the failure of that component in certain conditions. Image segmentation is a powerful technique for determination of morphological features of the microstructure like volume fraction, inclusion morphology, void, and crystal orientations. These are some key factors for determining the physical properties of metal. Therefore, automatic micro-structure characterization using image processing is useful for industrial applications which currently adopts deep learning-based segmentation models. In this paper, we propose a metallographic image segmentation method using an ensemble of modified U-Nets. Three U-Net models having the same architecture are separately fed with color transformed imaged (RGB, HSV and YUV). We improvise the U-Net with dilated convolutions and attention mechanisms to get finer grained features. Then we apply the sum-rule-based ensemble method on the outcomes of U-Net models to get the final prediction mask. We achieve the mean intersection over union (IoU) score of 0.677 on a publicly available standard dataset, namely MetalDAM. We also show that the proposed method obtains results comparable to state-of-the-art methods with fewer number of model parameters. The source code of the proposed work can be found at https://github.com/mb16biswas/attention-unet.

## Introduction

Manufacturing and core engineering domains are pioneering the application and adoption of Artificial Intelligence (AI)^1^. The primary objective here is to provide additional assistance to experts in those fields, consequently improving the effectiveness of the entire process. The characterization of materials that material scientists use or study to learn more about metals is an important factor in material science^2^. To be more specific, material characterization is a primary step in materials science, without which no scientific understanding of engineering materials can be achieved. Material scientists nowadays have access to a diverse range of scientific technologies that enable this characterization procedure. For material characterization, techniques such as X-ray, neutron, electron diffraction, light optical microscopy, and electron and ion beam microscopy are being used. However, some of these said methods are costly and time-consuming. Researchers frequently utilize optical microscopy, a technique that, when combined with other scientific methods and chemical processes, allows the composition and structure of a material to be ascertained. It is the procedure of viewing the structure on a much microscopic scale as revealed by an optical microscope at magnifications greater than $25\times$. This very small-scale structure of a material is often referred to as the material’s microstructure.

**Figure 1:**
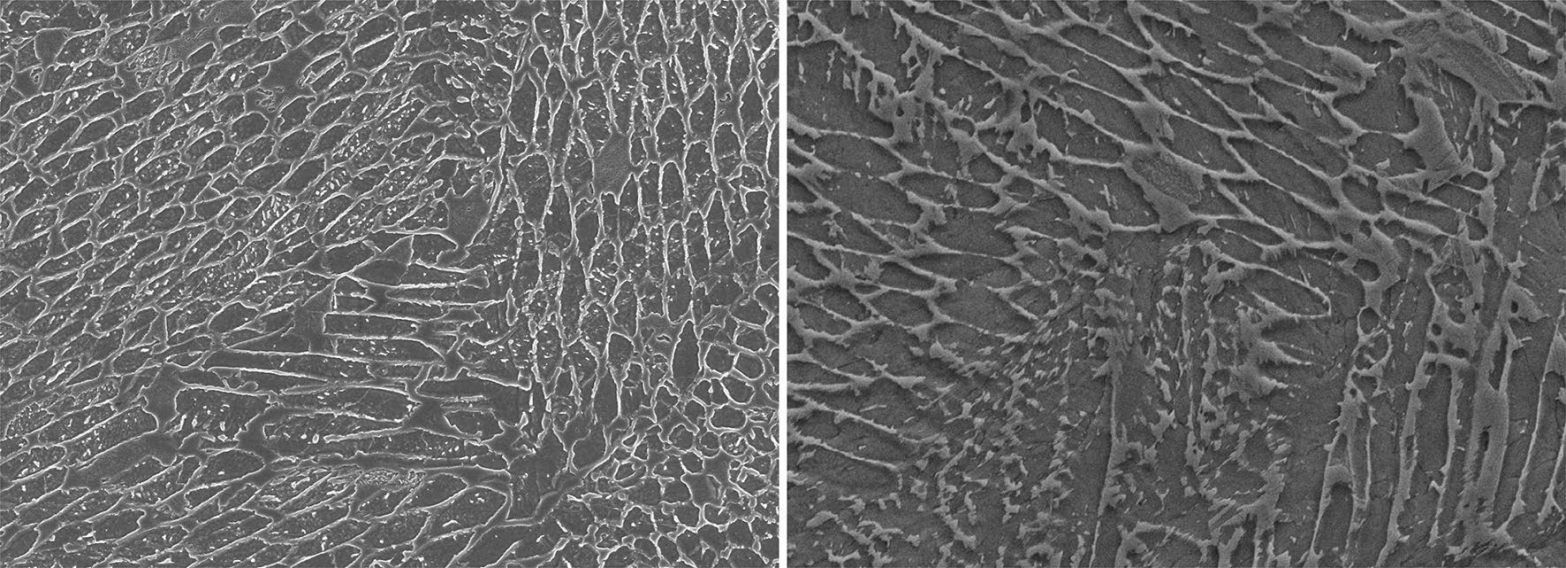
Microstructural images from metallographs. The images are taken from MetalDAM dataset^11^.

It is a well-known fact that the macroscopic mechanical properties of any material can be determined by its microstructure^3^. The microstructure of engineering materials is described by the grain size, types of phase present, and a description of their structure, shape, and size distributions^4^. In addition to that, two-dimensional defects such as grain boundaries and heterophase interfaces, one-dimensional defects such as dislocations, and zero-dimensional defects such as point defects are important microstructural features that often control the resulting properties^5^. Generally, microstructures are analyzed manually following the standard protocols provided by the American Society for Testing and Materials (ASTM). However, this method is time-consuming and labor intensive. Hence, as a viable alternative, currently many researchers are using AI techniques for the said purpose^6–8^. An AI-based microstructural analysis typically considers an image as the input to a model and retrieves information from each part of the image to produce the segmented image. Image segmentation is a digital image processing technique which is widely used in the fields of engineering, medical image analysis, computer vision, etc^9^. for identifying the distinct regions or zones in the image that contain recognizable visual attributes^10^. An illustration of metallographic images is presented in Fig. 1. The scanning electron microscope was used to observe these metallographic images. The scanning electron microscopy method uses an electron beam on the surface of a sample, the atoms and electrons interact with other to different types of signals of the surface morphology and composition information. The image is produced by adding the scanning path of the electron beam with the intensity of the detection signal.

In recent times, image segmentation using deep learning-based techniques has become very popular^12^. Among other deep learning models, U-Net based architectures are frequently used by researchers for many related tasks^12^. In a typical U-Net, there is an encoder and a decoder. The encoder is responsible for extracting the most informative feature representations within its hidden layers. The decoder, on the other hand, is tasked with interpolating the discriminative latent code to produce the segmentation map. Traditionally, researchers use a regular Red Green Blue (RGB) image, which is very useful to understand the visual context description. Additionally, to exploit chrominance-based information by intensifying the visual description, researchers in the past have explored color spaces such as hue, saturation, value (HSV) and light intensity, two color channels (YUV)^13^, where Y stands for brightness, U is the blue projection and V represents the red projection. It is quite obvious to note that all these color spaces illustrate complementary information, which can be very useful to learn via an aggregation technique. An ensemble network, in general, aims for the same, where the ensemble process focuses on learning to aggregate the potential complementary information^14^. To this end, in this paper, we propose an end-to-end pipeline for semantic segmentation of metallographic images. At first, after resizing the input images, we augment them using some standard image augmentation techniques described later. The images are converted into HSV and YUV. After that RGB, HSV and YUV versions are fed into the proposed U-Net based architecture separately. The confidence scores from the three U-Net models are combined to get an ensembled result. The proposed method has been evaluated on a publicly available benchmark dataset, called MetalDAM^11^. The primary **contributions** of this work are given below:

- We propose an end-to-end method for microstructural segmentation using an aggregation of U-Net models, where input images to the models are different in terms of color space.
- We propose an attention-guided U-Net model, which comprises channel and spatial attention modules to learn discriminative embeddings. We use dilated convolutions in the U-Net to reduce the chance of learning repetitive features.
- The proposed method achieves satisfactory results when evaluated on a publicly available metallographic image dataset.

The paper is organized as follows: Section Related work surveys deep learning-based methods proposed by other researchers for segmentation of metallographic images. Section Methods and materials presents a detailed description of our proposed approach, and Section  Results and analysis discusses experimental results. Finally, Sect. Conclusion states some concluding remarks about this work.

## Related work

Segmentation of metallographic images is considered a challenging task in the context that the final segmented image will impact the conclusive assessment of the material at the microstructural level. The rise of deep learning in recent times has triggered research in this domain. We review some of the recently proposed methods below.

The work by Azimi et al.^15^ used VGG-16 based encoder-decoder architecture, namely max-voted fully convolutional neural networks (MVFCNN). At first cropped patches of the original images were extracted, which were fed to the encoder-decoder model. In the process of cropping, a specific pixel was a part of multiple such patches. For the final pixel level classification, the help of majority voting was taken to determine the most appropriate class. This approach showed considerable robustness over its predecessors. However, one major problem with this method is the enormous amount of computation required to train VGG-based networks, thereby not making it suitable for real-time use. This is in addition to the amount of time required to employ a method using patches of the input samples. Lin et al.^16^ proposed a 3D convolutional neural network (CNN) based segmentation network for the detection of casting defects to extract microstructural properties. The casting defect region (CDR) CNN was designed to produce three objectives for each CDR: a class, a 3D semantic instance and a bounding box-based regressor (similar to Mask R-CNN^17^). A 3D Region Proposal Network (RPN) was made up of 4-layer deep CNNs, and the proposals were evaluated based on ground truth class level bounding boxes for each CDR. This RPN was used to extract geometric features which was based on 2D CDR maps supported by a ResNet50 backbone network. The 3D Instance Segmentation Network (ISN) was made up of ResNet 34 based backbone. In addition, a 3D Region of Interest (RoI) Align network was proposed for eliminating misalignment issues for the geometric features and the RPN. To test the network, the authors collected 8000 data samples which were manually annotated to decide bounding boxes and segmentation masks. The work provides a deep insight into multiple problems using a single pipeline. But, it uses a huge number of manually annotated data for the study. Additionally, the computation overhead is considerably large for real-time deployment. However, the method is carefully engineered to cater multiple problems to produce state-of-the-art results.

Roberts et al.^18^ proposed a DenseNet based U-Net model for semantic segmentation of metallographic images. Jang et al.^19^ proposed a ResNet-based U-Net segmentation network. Both of these approaches are well-known in the literature on semantic segmentation^12^. These are some of the earliest deep learning based methods used for metallographic segmentation. However, the authors provide a deep insight pertaining to these approaches for metallographic segmentation. Albuquerque et al.^20^ proposed Self-organizing map neural networks and Multi-layer Perceptron (MLP) based network for segmentation of the gray cast iron. The fusion of supervised and unsupervised models was found useful. This approach is computationally much cheaper than its deep learning counterparts, but having a low-dimensional latent space it does not let the model to learn discriminative features, provided by the CNNs. On the other hand, CNN-based segmentation networks produce a high-dimensional feature vector which is useful but computationally very expensive. Thus, maintaining this cost to usefulness is a challenging trade-off which is certainly an important factor while designing any AI-guided techniques.

Chen et al.^21^ investigated Aluminium alloy segmentation with different loss functions. The candidate loss function used in this work were binary cross-entropy, Dice loss, Intersection over Union (IoU) loss and a hybrid loss of sensitivity and specificity measures. The work by Ma et al.^22^ used the popular DeepLab-based segmentation network considering image level patches. Both of these methods were used to evaluate the Aluminium alloy defect detection using binary masks.

It is to be noted that currently many researchers are using the concept of semi-supervised approach for metallographic segmentation. Chen et al. ^23^ proposed a semi-supervised segmentation technique based on self-paced learning^24^. The formulation of pseudo labels for semi-supervision was based on the popular idea of expectation-maximization. A joint learning objective of supervised and pseudo labeled unsupervised algorithm was proposed by the authors. The whole idea was implemented on a basic U-Net. Unsupervised methods such as the one proposed by Kim et al.^25^ were based on mimicking metallurgists to detect defects. In this work, the super-pixels were firstly segmented out along with pixel wise classification using a CNN as a feature extractor. Then the segmented super-pixels along with the classified pixels were used to form a refined feature map for final segmentation

### Motivations

From above study, we observe that the literature of segmentation of metallographs is quite diverse. Several researchers attempted to tackle each of the unique challenges in this domain. The research articles^15,18,19^ provide a deep insight into this domain with relatively simpler yet effective methodologies. Further, the method by Lin et al.^16^ give a detailed study about microstructural properties with one of the disadvantages being the huge computational overhead. The semi-supervised learning approach^23^ has also been explored in this field. However, majority of the methods do not take any advantage of multiple predictions to exploit the complimentary information from multiple learners to generate better segmented masks. Ensemble learning is a field which aims for the same. Notably in various domains, the ensemble learning approach proves its worth^26–28^. However, a major problem with ensemble based methodologies is the increased computational overhead. To this end, in this work, we aim to design an ensemble of lightweight classifiers to take the advantage of both ensemble learning at the same time keeping in mind the computational overhead.

## Methods and materials

### Dataset description

The proposed method is evaluated on a publicly available dataset called MetalDAM, which is a metallography dataset from additive manufacturing of steels^11^. This dataset contains 42 labeled images with resolutions 1280$\times$895 and 1024$\times$703. The images in the labeled dataset are annotated pixel-wise according to the 5 micro-constituents present which are enumerated below:

1. **Matrix**: The metal matrix composite can offer better properties such as higher strength-to-weight ratios, stiffness, and ductility than traditional materials. Therefore, they are regularly used in critical engineering applications.
2. **Martensite/Austenite (MA)**: It is an exceptionally hard phase of steel. However, it also makes the metal more brittle^29^ and it cannot be welded or easily formed into other shapes^30^.
3. **Precipitates**: It increases the yield strength of the metal^31^.
4. **Austenite**: The presence of high austenite enhances the ability to be formed and welded easily into any shape along with providing great strength^32^ and resistance to corrosion^33^.
5. **Defects**: Defect formations are closely related to surface roughness, volumetric porosity and macro or micro-level cracking.

In this work, we deal with a semantic segmentation problem. Each pixel in an image of the said dataset belongs to one of the 5 classes/labels as mentioned above. In order to present a distribution of the classes over every image, we calculate the ratio of the total number of pixels belonging to a particular class to the total pixels of the images in the dataset. The distribution is presented in Table 1

**Table 1:** Pixel-wise class distribution considering all images of the MetalDAM dataset.

Index	Class	Distribution (%)
0	Matrix	31.86
1	Martensite/Austenite (MA)	8.96
2	Precipitates	0.24
3	Austenite	58.26
4	Defects	0.68

### Overall workflow

**Figure 2:**
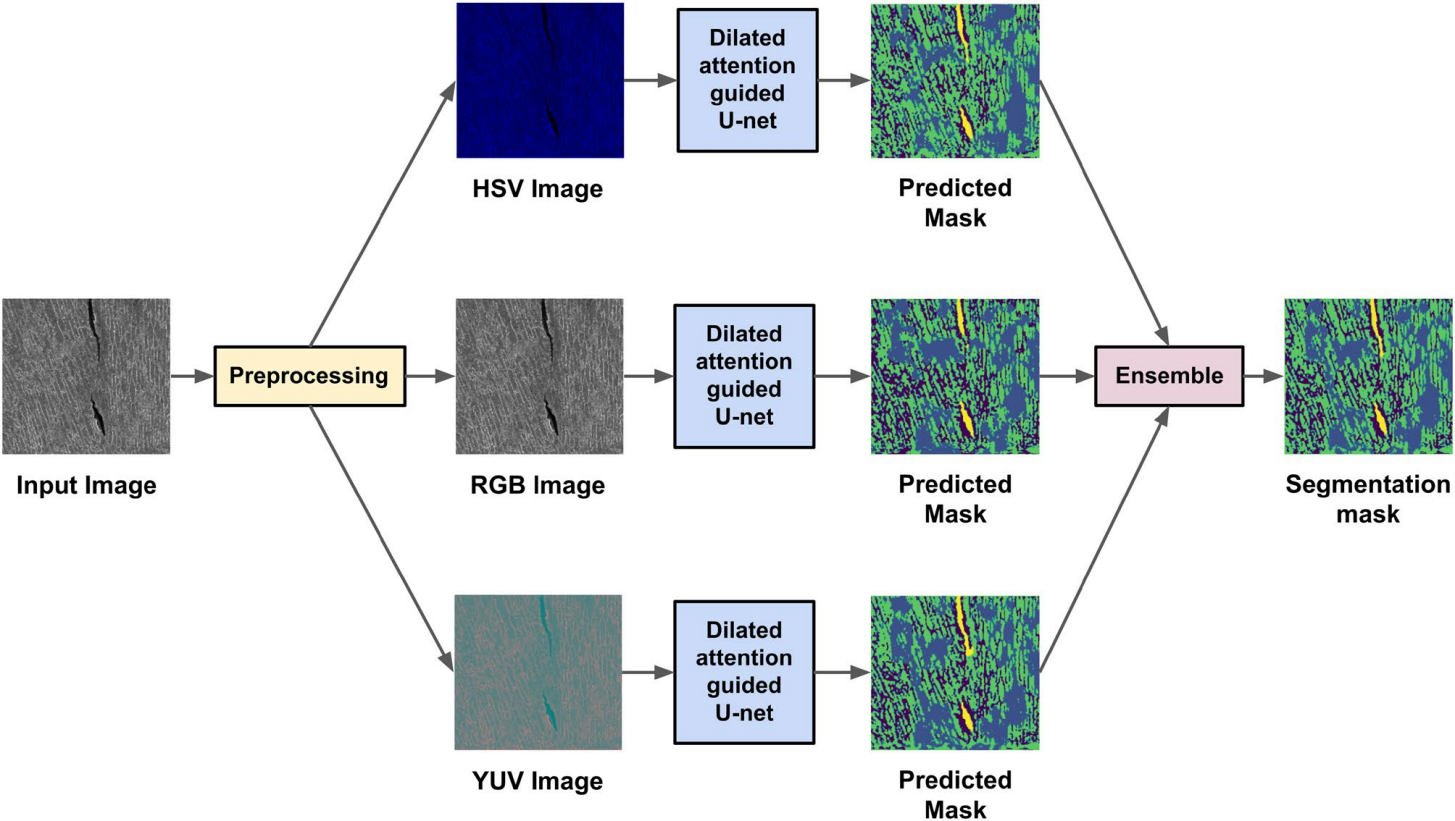
Overall pipeline of the proposed microstructure segmentation model.

We propose an ensemble network for microstructure segmentation from meallographic images. First, we transform the original RGB image into two other color spaces, namely HSV and YUV. We then feed the original image along with the transformed images to three separate (with the same architecture) attention-guided dilated U-Net models to obtain the segmentation masks. We then perform a pixel-wise addition to obtain the final prediction mask. Lastly, the respective predicted class is termed the final mask. An overview of the entire pipeline can be seen in Fig. 2.

### Image preprocessing

In this section we discuss some preprocessing techniques applied to metallographic images. It is noteworthy that the methods we discuss here do not require any kind of learning process or in simple words, these methods are static and do not require any additional data to produce the optimal values. We first resize the images to $(256\times 256)$ pixels to ensure the deep architecture performs in an unbiased manner^34^. Following standard process. As the pixel values of original images range from 0 to 255, high numeric pixel values will require more computational power. Hence, we follow the image normalization process by normalizing the pixel values to range from 0 to 1 by dividing each pixel value by the highest possible pixel value i.e., 255.

Once we apply the pre-processing techniques on all the samples, we augment the training samples to make the CNN models more competent to handle translated input samples^35^. To avoid evaluating on synthetic data, we refrain ourselves from augmenting the test and validation samples. To be specific, we adjust the brightness, use gamma correction, adjust hue, crop, flip it horizontally and vertically and rotate it by ninety degrees. We perform these operations to increase the competence of our CNN architecture and to increase the number of labelled samples, considering the fact that the dataset used here is relatively very small in size^36^. To perform the first three augmentation techniques, where the ground mask was left untouched. On the other hand, the rest of the techniques require the ground mask to be transformed in a similar manner.

#### Chrominance transformation

We consider two chrominance-based transformations along with the RGB image, and feed these to three separate U-Net based segmentation models having the same architecture. In this work, we employ two very popular chrominance-based transformations, namely HSV and YUV. We introduce an approach that is made up of different color spaces to neutralize the effects of lighting conditions^37^. In HSV space, the H determines the colors ranging from 0 to 360 degrees. The S & V channels contain information about saturation and intensity properties, respectively. The YUV color space is purely based on luminosity. Here, Y is an equalized channel. U is positive only if blue is greater in comparison to certain percentage of red and green. V is positive only if the red is greater compared to a certain percentage of blue and green^38^.

**Figure 3:**
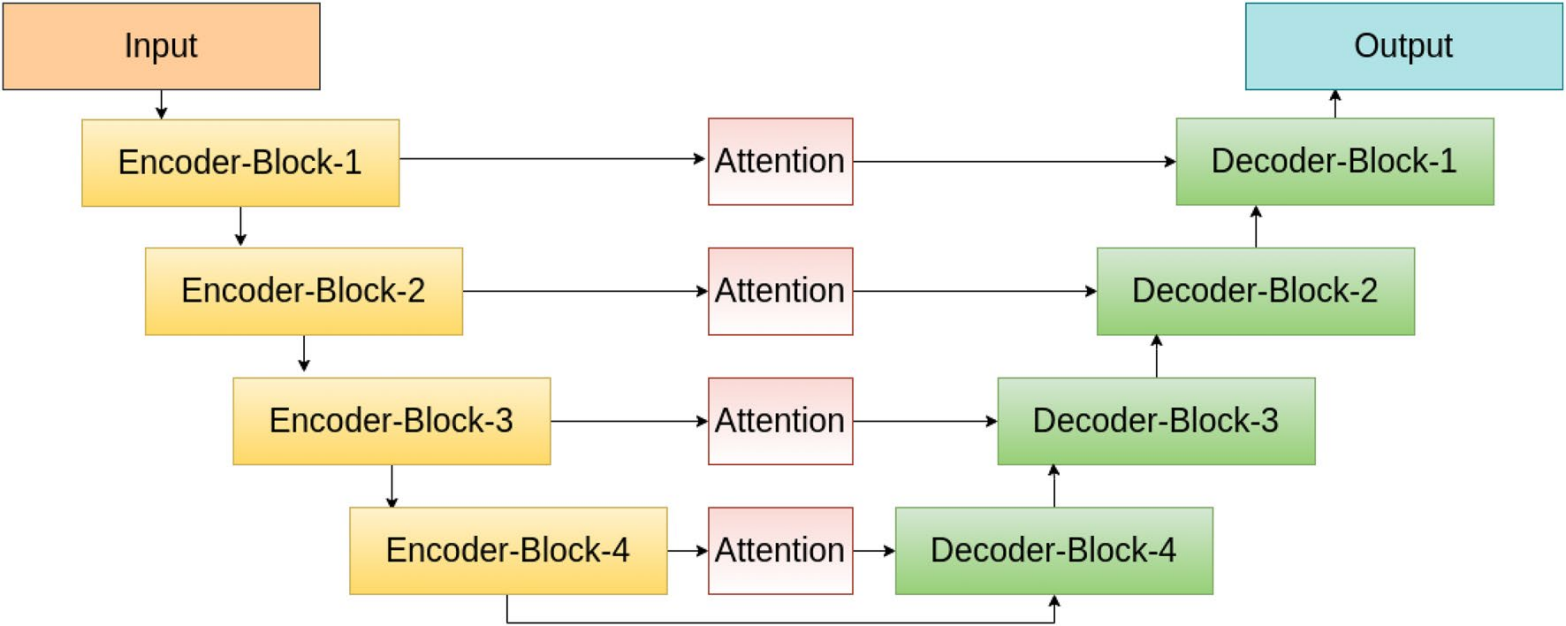
An illustrative diagram of the U-Net model used in this work.

Thus it is very well-perceived from the above discussion that RGB, HSV and YUV color spaces can produce various potential information distinct to each other which we finally aggregate to acquire more discriminative information i.e., segmentation masks. Thus, in this work, the U-Net models intend to learn complimentary information from these color spaces of the same input. After obtaining the segmentation maps from RGB, HSV and YUV color spaces using the proposed U-Net models, we apply the sum rule to obtain the final segmentation map. An illustrative diagram of the proposed U-Net model is given in Fig. 3.

### Dilated U-Net

We compose the proposed network architecture with a contracting path (encoder) and an expansive path (decoder). The architecture has 4 blocks in each of the paths. We use an encoder to reduce the spatial dimensions of the inputs in every block, at the same time to increase the temporal dimension. Consequently, we use a decoder to increase spatial dimensions while decreasing the temporal dimension. As opposed to the original U-Net architecture described by Ronneberger et al.^39^, in this work, in each block we consider two levels of convolution with dilated filters^40^. The use of dilated convolutions allows us to capture multi-scale global information without increasing the filter size. This reduces the chances of overfitting by leaving out certain repetitive features. At the same time, this allows the network architecture to have a lesser number of trainable parameters. We sequentially reduce the dilation factor from 4 to 1 for the four blocks.

In each block, convolution is followed by batch normalization to re-centre and re-scale the features, which helps to regularize the overall architecture. We further apply rectified linear unit (ReLU) based activation and a 2×2 max pooling operation with a stride of 2 is performed to reduce the spatial dimensions in the encoder. The decoder consists of an upsampling of the feature map with bilinear interpolation. We stack these interpolated features with the corresponding feature map from the encoder. Next, we apply two levels of convolution (with 3×3 filters) followed by batch normalization and ReLU-based activation. The layers in the encoder part are skip-connected and concatenated with layers in the decoder part. To enhance the quality of the encoder features used in the decoder network, we introduce an attention block (see Fig. 3). This makes the U-Net learn fine-grained details of the encoder to construct an image in the decoder part. At the last layer, we use a convolutional layer with a 1×1 filter and the number of filters is equal to the desired number of classes.

### Attention module

In human perception, attention plays a significant role^41^. It is also noted that as humans while attempting to pay attention (focus) to a visual scene, they do not try to process the whole scene at once^42^. Inspired by this natural human instinct, Woo et al.^43^ proposed a simple yet effective attention mechanism consisting of channel and spatial attention modules.

**Figure 4:**
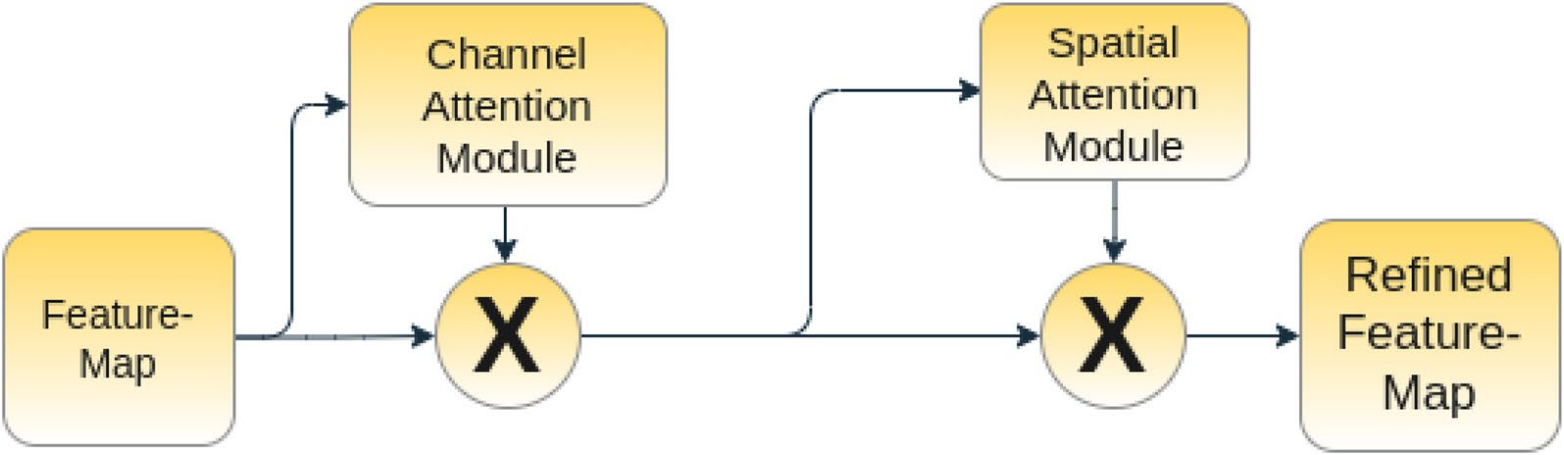
An illustration of the attention modules used in this work.

We apply attention to skip connections between the encoder and the decoder in the U-Net (see Fig. 3). We use both average and maximum feature aggregators to pool the features in order to generate the attention maps for retrieving finer-grained discriminative features. We first use the channel attention module followed by the spatial attention module (see Fig. 4). We describe each of the modules in detail below.

#### Channel attention

**Figure 5:**
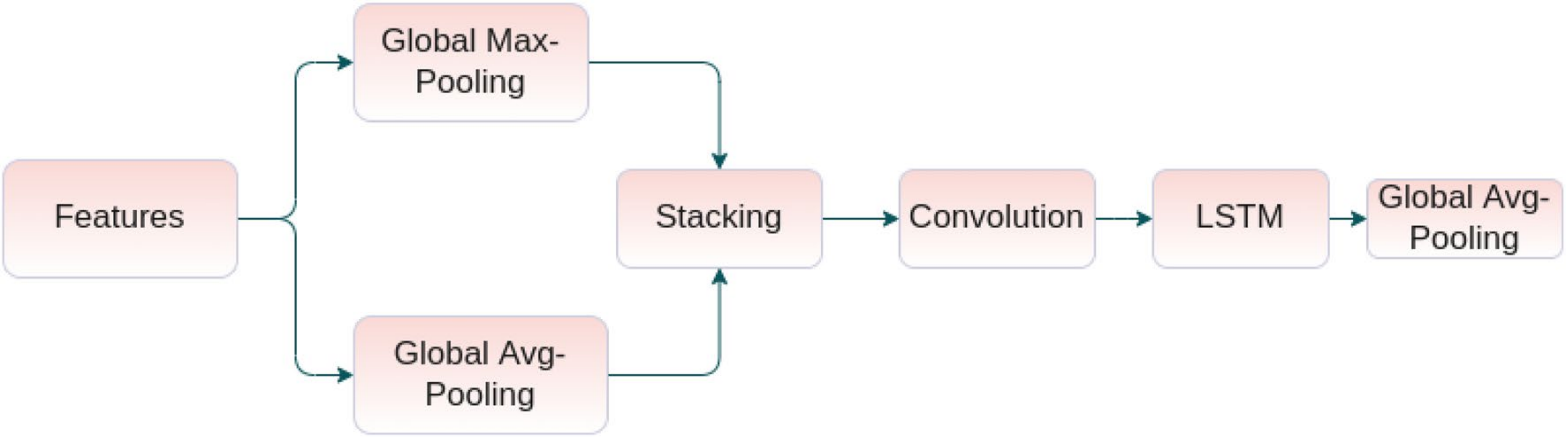
An illustration of the channel attention module used in this work.

Each channel in a classical CNN is known as a feature detector^44^, while each channel corresponds to ’what’ is meaningful in the given features. To compute channel attention scores, we first obtain the pooled features by applying global average pooling and global maximum pooling. We then stack these pooled features and apply 1D convolutions having kernel size 1, with 32 filters followed by a long-short term memory (LSTM) based embedding (with the number of cells equal to 64) to learn inter-channel dependency. Considering the fact that LSTM is widely used to capture the long and short range dependencies among the features in the natural language processing (NLP) domain. The main idea is to capture inter-feature relationships and embed the importance scores to have a better localization ability. This is achieved by determining long range temporal dependencies among the channels which, in turn, enhance the localization capability of the U-Net architecture. We perform an average pooling operation within these features to generate the attention scores. Finally, we multiply these attention scores by each of the features in the corresponding channels to produce the channel attentive features. We provide a diagram for the channel attention module in Fig. 5.

#### Spatial attention

**Figure 6:**
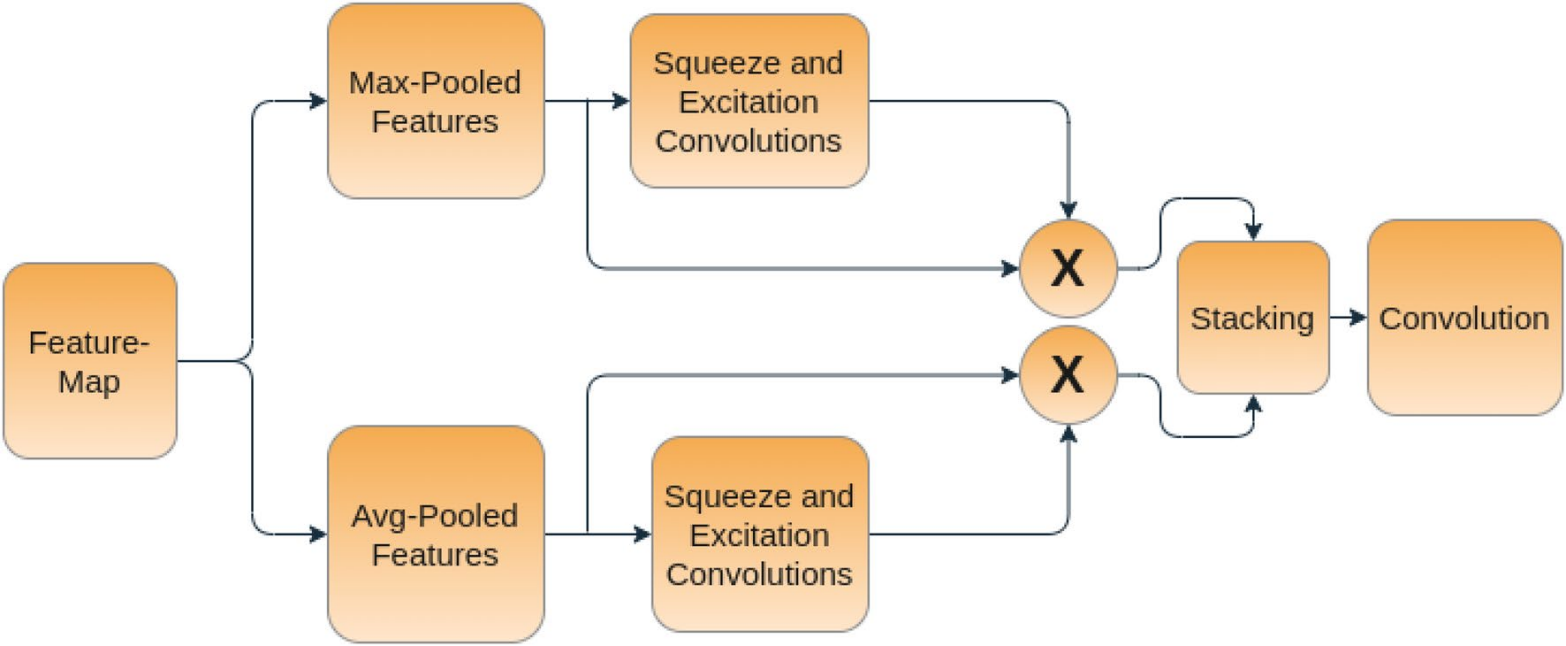
An illustration of the spatial attention module used in this work.

To generate the spatial attention map, we focus on the inter-spatial relationship among the features. We apply pooling along the channel axes to generate the pooled features. It is noteworthy that in literature studies have shown that such strategies are effective in highlighting informative regions^45^. The pooled feature maps are then processed using a convolution-based squeeze and excitation network, where we first increase the number of channels while maintaining the spatial dimension and then decrease the number of channels to 1. Then, these features are stacked, and one more level of convolution (with filter size 7×7) is applied to generate the final attention map. This attention map is applied to every element in the spatial dimension. The diagram of the spatial attention module used in this work is given in Fig. 6.

### Loss function

In this work, we have used a hybrid loss function which is described below.

**Focal loss**: The class imbalance problem creates bias while we use the Cross-Entropy loss, i.e., the majority class influences the loss value resulting in the model becoming more confident in predicting the majority class samples only. In such scenarios, Focal loss works better on class imbalance datasets during the training process. Focal loss overcomes this by down weighting, i.e., by reducing the influence of easy-to-train samples for the loss, resulting in more attention being paid to the hard-to-train samples. This is achieved by adding a modulating factor to the Cross-Entropy loss to focus learning on hard misclassified samples. It is dynamically scaled, where the scaling factor decays to zero as confidence in the true positive class increases. Subsequently, this scaling factor can automatically down-weight the contribution of easy samples during training and rapidly focus the model on hard samples. The formulation of Focal loss is given in Eq. (1), where $\gamma$ is the modulating factor.

1 $$\begin{aligned} Focal \, loss = - (1-p_{t})^\gamma \times \log p_{t} \end{aligned}$$

**Dice loss:** Dice loss directly optimizes the Dice coefficient, which is one of the most widely used evaluation metrics for segmentation tasks. The Dice coefficient is a measure of overlap between two samples. This measures the range from 0 to 1, where the Dice coefficient of 1 denotes perfect overlap. Equation (2) presents the formulation of Dice loss, where $|A \cap B|$ represents the common elements between sets A and B, and |*A*| represents the number of elements in set A.

2 $$\begin{aligned} Dice \, loss =1-\frac{2 \times \left| A \cap B \right| }{\left| A\right| + \left| B\right| } \end{aligned}$$

**Hybrid loss:** In this work, we have hybridize the above-mentioned two different loss functions having different training dynamics to show different properties. This helps to combine the advantages of the said loss functions while reducing their disadvantages. The hybrid loss function is defined in Eq. (3).

3 $$\begin{aligned} Hybrid \, loss = Dice loss + Focal Loss \end{aligned}$$

## Results and analysis

In this section, we first discuss the evaluation criteria and the experimental protocols used here. Next, we present the experimental findings and analyse the same.

### Evaluation techniques

In this work, we resort to the 6-fold cross-validation technique to assess the performances of our method. For this, we split the original samples into 6 fractions. At each run, we consider one fraction for testing and the rest for training and validation. Out of the five fractions of data used for training and validation, one fraction is randomly separated out for validation. We have followed this approach because the dataset considered here is relatively small in size. However, validation is required to make sure that our method does not overfit the training data.

### Evaluation metrics

Accuracy is the simplest metric to evaluate the performance of the model. In our case, the accuracy represents the percentage of pixels in the image that are correctly classified by comparing each pixel one by one with the ground truth mask. Despite showing a general overview of the model’s performance, accuracy scores are biased whilst dealing with imbalanced datasets. It should be kept in mind that we deal with a heavily imbalanced dataset (see Table 1). Out of 5 classes, 2 classes with class indices 0 & 1 consist about 90.12% of the total predictions. Therefore, it is important to judge both the performance of the majority and minority classes.

To deal with an imbalanced dataset like ours, we also consider the F1 score metric. The F1 score is evaluated as the harmonic mean of the precision and recall scores. The precision score signifies the ratio of the total correctly classified positive instances to the total positive instances. Contrary to this, the recall score measures the coverage of minority class^46^.

The IoU metric is also referred to as the Jaccard index. It quantifies the percentage of overlap between the target mask and the prediction output. In simple words, the IoU metric measures the ratio of the number of pixels common between the target and prediction masks and the total number of pixels present across both masks. IoU is a well-known performance metric used for image segmentation problems^12^ due to its ability to effectively capture the degree of overlap between the ground truth and the predicted mask.

### Experimental results

In Table 2, we observe the results of the proposed method using 6-fold cross-validation scheme. We can observe that the values obtained for all three color spaces are more or less similar from an overall perspective. However, if we go through the data minutely, we can see that in some of the folds the values are suboptimal for some color spaces, but for other color spaces we find better results for the same split of data. This is a clear indication of presence of complimentary information, which we intend to aggregate here by using an ensemble method. The results obtained post-ensemble are significantly better than its base counterparts. Thus, the use of ensemble learning in this scenario is justified. While observing the results class-wise, we observe that the use of ensemble learning helps to overcome some of the shortcomings of the base models to a large extent by using an aggregation of the color spaces. Therefore, we can claim that the use of an ensemble of different color spaces helps the model to produce better results.

**Table 2:** Results obtained using the 6-fold cross validation scheme for the main pipeline. Each IoU score is for a particular type of pixels as mentioned in Table 1.

Fold	RGB					HSV					YUV					Ensemble				
	IoU0	IoU1	IoU3	IoU4	Mean IoU	IoU0	IoU1	IoU3	IoU4	Mean IoU	IoU0	IoU1	IoU3	IoU4	Mean IoU	IoU0	IoU1	IoU3	IoU4	Mean IoU
1	60.28	51.97	72.45	64.28	62.24	58.34	49.59	71.56	65.51	61.25	60.63	50.01	71.02	67.81	62.37	64.17	58.39	74.94	72.54	67.51
2	57.97	50.70	70.72	69.26	62.16	59.57	50.89	72.43	70.48	63.34	60.24	51.73	72.86	51.47	59.07	62.97	59.43	74.63	73.44	67.62
3	57.56	49.57	72.92	76.27	64.08	57.89	51.15	73.03	74.83	64.22	58.27	48.51	73.31	77.00	64.27	61.59	58.15	75.48	79.52	68.69
4	57.69	51.99	70.34	66.01	61.51	54.63	41.79	65.68	63.04	56.29	55.94	51.70	70.50	69.56	61.93	60.84	58.99	72.44	72.14	66.11
5	58.18	53.38	72.49	67.58	62.91	58.68	50.51	73.75	72.29	63.81	59.33	56.31	73.65	74.60	65.97	62.78	60.27	76.24	78.30	69.40
6	56.38	45.79	72.88	78.03	63.27	51.55	40.73	70.79	68.85	57.98	58.41	47.82	72.90	72.62	62.94	60.37	53.67	75.21	79.97	67.31
Average	58.01	50.57	71.96	70.24	62.70	56.78	47.44	71.21	69.17	61.15	58.80	51.01	72.37	68.84	62.76	62.12	58.15	74.82	75.99	**67.77**
Std Dev	1.28	2.67	1.14	5.63	0.92	3.07	4.83	2.90	4.35	3.32	1.69	3.05	1.29	9.14	2.33	1.44	2.32	1.29	3.66	1.15

Significant values are in bold.

**Figure 7:**
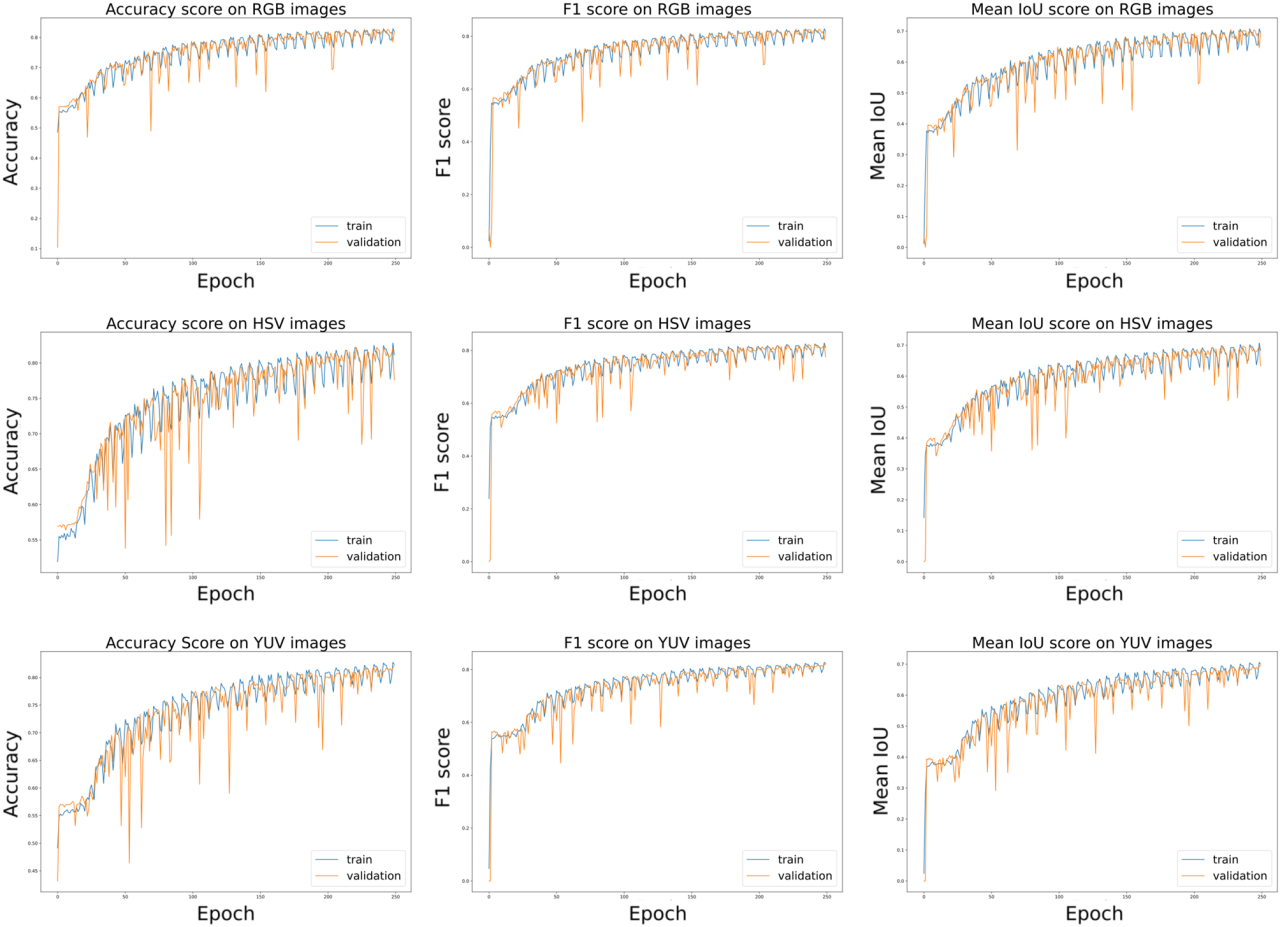
Learning curves w.r.t to accuracy, F1-score and mean IoU score concerning the RGB, HSV and YUV based base models on Fold-1 of the experiment.

In Fig. 7, we present the learning curves concerning Fold-1 of the experiment. From the curves we can see that the model does not suffer from any major overfitting. We note that there is a bit of instability observed in the training process which can be attributed to the fact that the dataset used in this work is very small in terms of the number of samples.

**Figure 8:**
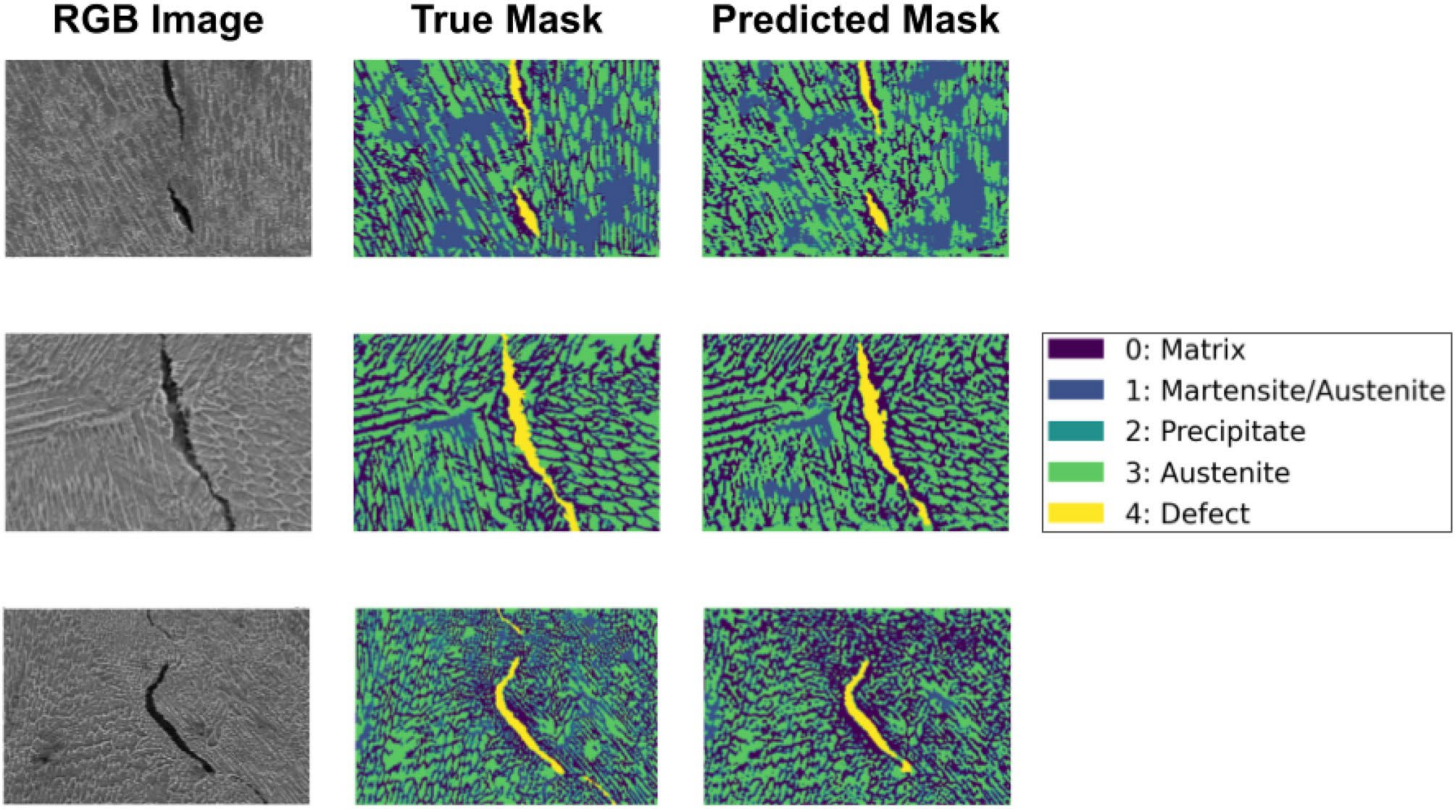
Comparison among original, ground-truth and predicted images. Here, different colors represent different classes.

In Fig. 8, we present the true mask and predicted mask using the proposed method for three different samples. From the figure, we see that the predicted masks are considerably good when compared against the ground-truth mask. We see that in the boundary prediction, the method overestimates a bit. One strong possible reason can be that these pixels are much less compared to other classes. However, this shortcoming might be avoided if we use a larger dataset to train our model. Among all the categories present in the microstructure, each category contributes to different properties. It can be observed from Table  2 that the ’defect’ and ’martensite/austenite’ classes have higher mean IoU score than others. The class ’defect’ leads to the formation of micro- or macrolevel cracking formation. It is an indication of material failure that can ultimately lead to complete failure. The presence of ’martensite/austenite’ class makes the metal brittle that means when subjected to stress, it fractures with little elastic deformation. The other classes are also equally important and have applications in engineering domains. The matrix class improves mechanical and thermal properties that embrace good wear resistance and exceptional thermal conductivity. The other segmented class is Austenite, which gave good formability and weldability along with excellent toughness. The final segmented class is Precipitates, this creates a harder, stronger metal.

### Time analysis

In this work, we propose an ensemble-based segmentation methodology. Thus, it becomes an important factoTo train each of the modelr to discuss the time required to train this methodology for the end-to-end segmentation task. To conduct the experiments, we used machines with RAMs extending up to 28 GB powered by a single Nvidia Tesla T4 GPU. The programming language used to implement is Python 3.9 along with support from Tensorflow 2.8.2 and Keras 2.8.0 library to implement the deep learning models.

It should be kept in mind that for each of the three U-Net models, the time required to train them is the same. To train each of the models, we require 3s per epoch on the standard 6-fold cross-validation scheme as described above. We train each of the networks for 250 epochs, therefore, the total time required to train the model is 12.5 min for each model. Given that we have three such models, we require 37.5 min to train the model for each fold. To perform the 6-fold cross-validation, we require a total time of 3 hours and 45 min. To obtain the inference time, the U-Net model has been run with a batch size of 1. The model has been run for 100 times, and the average time of all forward passes is taken as its inference time. The model takes 0.046s on an average for each forward pass with a standard deviation of 0.007s.

### Statistical test

We conduct a statistical test to measure the robust nature of the proposed ensemble method w.r.t. the base models. We hypothesize *“The ensemble network produces similar results when compared to the base models”*. To perform this test we take the help of a very popular non-parametric statistical test, namely the Wilcoxon Rank-Sum test^47^. We compare the mean IoUs of the base models and the ensemble model fold-wise to conduct this test. The obtained results are tabulated in Table 3.

**Table 3:** Results of the Wilcoxon Rank-Sum test.

Base model	*p* value
RGB	0.03125
HSV	0.03125
YUV	0.03125

From the results shown in Table 3, we can easily reject the null hypothesis for every case since the obtained* p* value is less than $0.05\;(5\%)$. We observe the obtained values are equal in magnitude. However, it does not impede the statistical test as the Wilcoxon Rank-Sum test is a rank based test, i.e., it does not depend on the magnitude of the results. Finally, we can also claim that the use of ensemble methodology in the present work helps to produce statistically significant results.

### Ablation study

We conduct an ablation study concerning the attention modules used in this work. In Table 4 we provide the results for U-Net without and with channel attention, respectively. From the results, it is clear that the use of attention highlights the discriminative regions of the feature maps. As we discussed in the previous section, the use of channel attention helps to learn what to focus on considering the fact that each channel is a separate feature detector. The use of spatial attention coupled with channel attention helps to learn more discriminative embeddings compared to both methods (see Table 2). Thus, we can safely claim the importance of using attention modules in this work.

**Table 4:** Results of the 6-fold cross-validation scheme for the proposed U-Net model without and with the channel attention on RGB images. Each IoU score is succeeded with the class index as mentioned in Table 1.

Fold	Without channel attention					With channel attention				
	IoU0	IoU1	IoU3	IoU4	Mean IoU	IoU0	IoU1	IoU3	IoU4	Mean IoU
1	58.30	43.71	72.12	65.55	59.92	52.91	45.90	67.53	63.83	57.54
2	56.51	46.92	70.80	67.16	60.35	59.46	50.02	72.76	60.83	60.77
3	53.11	41.00	70.50	74.46	59.77	57.20	50.81	72.21	71.50	62.93
4	47.57	44.23	65.00	53.88	52.67	54.02	52.00	71.09	62.72	59.96
5	57.67	49.21	72.60	77.25	64.18	55.06	50.25	69.08	76.31	62.68
6	40.21	26.55	59.64	50.70	44.28	55.87	49.63	71.93	75.77	63.30
Average	52.23	41.94	68.44	64.83	56.86	55.76	49.77	70.77	68.49	61.20
Std dev	7.10	8.05	5.10	10.70	7.20	2.34	2.06	2.04	6.88	2.22

In Table 5 we provide outcomes of the ablation study concerning different loss functions. From the table we see for the Dice loss, the model is unable to predict some classes while for the Focal loss, the results are considerably better. When we use the proposed hybrid loss, we see that the metrics are much better when compared to the two base losses. It should be noted that the use of Dice loss does not degrade the results for some of the classes, rather the use of it in conjunction with the Focal loss helps to scale up the results for all the classes significantly. Thus, as claimed in the previous subsection, our proposed hybrid loss helps to combine the advantages of the two losses considered here and compliments the disadvantages of the other counterpart loss.

**Table 5:** Results of the 6-fold cross-validation scheme using the proposed U-Net model on RGB images for different loss functions. Each IoU score is succeeded with the class index as mentioned in Table 1.

Fold	Focal loss					Dice loss					Hybrid loss				
	IoU0	IoU1	IoU3	IoU4	Mean IoU	IoU0	IoU1	IoU3	IoU4	Mean IoU	IoU0	IoU1	IoU3	IoU4	Mean IoU
1	35.66	48.08	60.9	52.94	49.39	59.69	0.00	72.96	0.0	33.16	59.42	52.7	73.28	63.35	62.19
2	41.61	38.91	58.26	55.12	48.48	59.01	0.00	71.94	0.00	32.74	59.89	50.24	71.15	67.50	62.20
3	46.86	44.64	63.65	66.02	55.36	58.43	0.00	72.63	0.00	32.77	59.26	47.99	71.65	75.96	63.72
4	35.26	39	57.8	37.46	42.38	55.63	0.00	70.63	0.00	31.57	56.6	53.46	71.82	67.27	62.29
5	34.66	31.57	52.97	58.01	44.30	47.96	0.00	67.14	0.00	28.77	59.12	55.31	73.25	70.11	64.45
6	34.33	43.16	60.36	58.32	49.77	55.47	0.00	71.18	0.00	31.66	55.94	46.08	71.80	78.86	63.17
Average	38.06	40.89	58.99	54.65	48.28	56.03	0.00	71.08	0.00	31.78	58.37	50.96	72.16	70.51	63.00
Std dev	5.08	5.75	3.62	9.52	4.57	4.33	0.00	2.12	0.00	1.61	1.66	3.50	0.89	5.84	0.94

### State-of-the-art comparison

We present the baseline state-of-the-art comparison for the MetalDAM dataset in Table 6. From the results in the table, it is very well observed that the proposed method gives competitively better results, with one of the methods yielding similar results when compared with the baseline models. Also, it should be noted that the U-Net and U-Net++ used in those experiments differ when compared to the present setup. The models described in the table contain much more parameters when compared to the present U-Net model. Artificial MultiView Ensemble (AMVE) and Stacking-based ensemble methodologies on this dataset with different architectures using the same image. The authors use EfficientNet B0 based encoder as the backbone architecture for all these architectures. The U-Net used in the previous work^11^ consists of 6,252,049 parameters compared to our model, which has only 597,637 parameters, which is less than $10\%$ of the one used in the previous work. We observe that our methods perform marginally better, this increase can be attributed to the fact that we use dilated convolutions to avoid learning redundant features. Additionally, the use of attention lets our model learn more finer grained features. We observe that our model has very few parameters than the baseline models, which eventually can be a deciding factor for deployment in resource constraint environments. Thus, our method has a significant edge over the pre-existing methods for real-time usage.

It is to be noted that the increase during the ensemble for the baselines for the previous work is very marginal when compared to our method. This is possibly because in the previous work, the authors consider ensemble learning using the same image, which is crucial, considering the fact that the main motivation to use an ensemble learning model is to learn complimentary information which in the case of the previous work is questionable. Thus, we can claim that our methods work at par in comparison to the past methods with very fewer trainable parameters.

**Table 6:** Performance comparison of the proposed method with state-of-the-art methods on the MetalDAM dataset. All other results are reported in the work of Luengo et al.^11^.

Method	Mean IoU	#Pramas
DeepLabV3+	61.37	4,908,497
FPN	58.96	5,760,001
U-Net	61.00	6,252,049
U-Net++	66.11	6,570,161
Stacking 2 best	67.47	12,822,890
Stacking 3 best	67.77	17,731,387
AMVE 2 best	61.85	12,822,440
AMVE 3 best	64.44	17,730,937
Proposed-UNet-RGB	62.70	597,637
Proposed-UNet-HSV	61.15	597,637
Proposed-UNet-YUV	62.76	597,637
**Proposed-ensemble**	**67.77**	1,792,911

Significant values are in bold.

### Additional tests

In Table 7, we present the results using 5-fold cross-validation settings. From this table we see that the proposed method produces a mean IoU slightly lower than the 6-fold cross-validation, as stated above. This is because for the exact reason that in the 5-fold cross-validation, the training samples are lower than in the 6-fold cross-validation setting. The more training data, the more local features are learned by the model. As the more local features are learned by the model, the more finer masks are produced. However, it is interesting to note that even with lesser training data, our ensemble methodology works well which further ensures that the use of chrominance transformations provides to models to learn complimentary information which later we have aggregated.

**Table 7:** Results obtained using the 5-fold cross-validation scheme for the main pipeline. Each IoU score is succeeded with the class index as mentioned in Table 1.

Fold	RGB					HSV					YUV					Ensemble				
	IoU0	IoU1	IoU3	IoU4	Mean IoU	IoU0	IoU1	IoU3	IoU4	Mean IoU	IoU0	IoU1	IoU3	IoU4	Mean IoU	IoU0	IoU1	IoU3	IoU4	Mean IoU
1	59.81	48.48	71.01	66.49	61.45	61.96	51.60	71.65	63.55	62.19	60.59	49.37	72.57	64.99	61.88	64.05	56.31	74.24	69.64	66.06
2	60.69	44.79	73.63	77.81	64.23	61.01	40.24	73.85	76.29	62.85	59.13	47.33	73.95	76.16	64.14	63.30	47.95	75.93	78.92	66.52
3	56.18	51.26	70.70	66.29	61.11	53.29	50.38	70.91	70.65	61.31	58.36	53.66	71.09	68.94	63.01	59.45	58.55	73.17	73.67	66.21
4	57.19	47.75	72.23	62.20	59.84	52.14	43.31	71.21	70.94	59.40	58.82	46.29	72.27	74.49	62.97	61.20	54.84	74.82	75.57	66.61
5	58.29	45.10	71.68	82.42	64.37	53.91	40.80	71.80	77.92	61.11	52.60	40.61	71.32	78.19	60.68	59.81	51.14	74.46	83.10	67.13
Average	58.43	47.47	71.85	71.04	62.20	56.46	45.27	71.88	71.87	61.37	57.90	47.45	72.24	72.55	62.54	61.56	53.76	74.52	76.18	66.51
Std dev	1.84	2.66	1.16	8.61	2.01	4.64	5.37	1.15	5.65	1.30	3.08	4.75	1.14	5.45	1.31	2.05	4.22	1.00	5.12	0.41

### Further analysis

**Figure 9:**
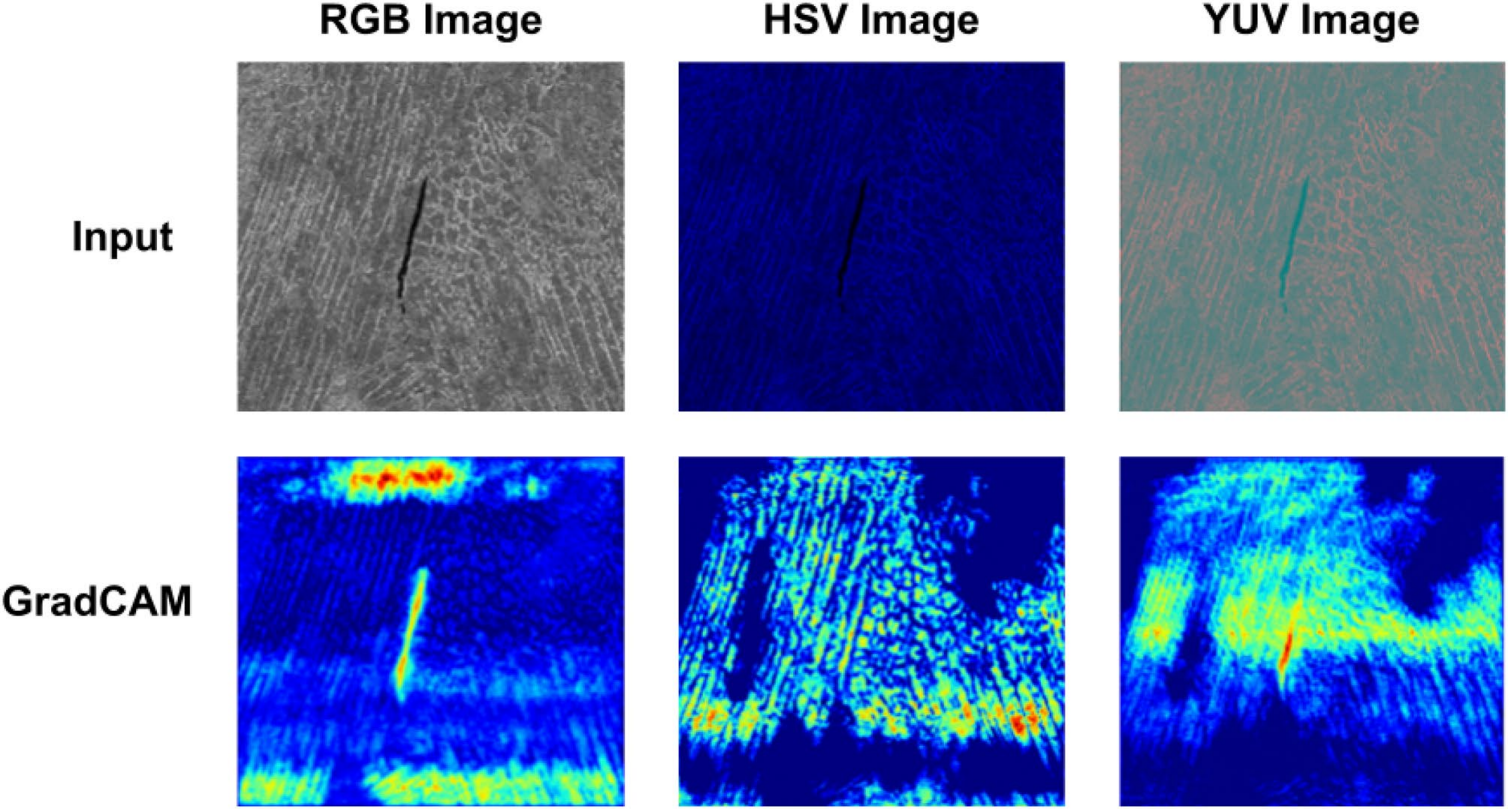
Input images in three color spaces and their corresponding GradCAM images.

It is important to note that in an ensemble based methodology, components, i.e., the base learners, are supposed to provide complementary information. To ensure this fact, we present the Gradient-weighted class activation maps (GradCAM) for all three base learners, where we feed different color models of input images. From Fig. 9, we can observe that the three U-Net models do not focus in the same way over the entire regions of the inputs. The RGB, HSV, and YUV images are visually different from each other, i.e., images in different color spaces look differently due to varied contrasting effects, which results in the focus on separate regions by the U-Net models, as seen in the GradCAM images. This is helpful for any ensemble framework. We also observe that the GradCAMs have slight to medium activation in areas, where other models have high activation, thereby indicating that all three base models do not provide completely complementary information, i.e., there are some overlapped regions focused by each of the three models.

## Conclusion

The metallographic images contain valuable information about the metal. It is possible to draw some important inference regarding the characteristics of the metal from those metallographic images. Image segmentation is a useful method that helps to analyse metallographic images for better understanding their properties. In this work, we propose a dilated U-Net based architecture with an attention mechanism for semantic segmentation of metallographic images. The proposed attention module learns fine-grained details from the encoder part of the U-Net. In the future, we may consider adding some kind of boundary attention mechanism like those shown by Zhao et al.^48^ to pay more attention in estimating the boudary regions.

The method proposed here has an increased training time because we need to train the U-Net model thrice for different color spaces. Hence, in future we plan to consider region proposal based architectures like Mask RCNN to generate region proposals within a single architecture for different chrominance transformations of the same image. The dataset used in this experiment is very small in size. In future, we will explore self-supervised learning (SSL) based learning methodologies to pre-train the network with both labeled and unlabeled data. Additionally, we wish to see new benchmark datasets much larger in size available publicly to advance the state-of-the-art research in this domain.

## Data Availability

The datasets generated and/or analysed during the current study are available in the GitHub repository, https://github.com/ari-dasci/OD-MetalDAM.
